# Exploring new scientific innovations in combating suicide: a stress detection wristband

**DOI:** 10.11604/pamj.2024.49.98.43956

**Published:** 2024-11-28

**Authors:** Daniel David Otobo, Raul Caballero Montes, Phuc Sheryl Vu, Vince Bigas

**Affiliations:** 1 The Mending Minds, Innovation for Action Global Health Challenge Team, Boston, Massachusetts, United States of America,; 2 Global Surgery Fellow, Operation Smile International, Virginia Beach, Virginia, United States of America,; 3 Department of Biology and Biochemistry, Houston, University of Houston, Texas, United States of America,; 4 Department of Cancer Biology, The University of Texas MD Anderson Cancer Center, Houston, Texas, United States of America,; 5 Department of Cell Systems and Anatomy, University of Texas Health Science Center at San Antonio, San Antonio, Texas, United States of America,; 6 Department of Philosophy, Eton College, Windsor, Berkshire, United Kingdom

**Keywords:** Stress, suicide, wristband, prevention, hormones

## Abstract

There is a silent pandemic of suicides around the world, with an exponential increase in suicidality and chronic suicidal ideations. The exact global estimates cannot be accurately ascertained, but analysis will put it at more than a million annually. With countries like America having almost 50,000 and India alone reaching 200,000, annually. Countries like Bangladesh are nearly chronically suicidal. However, in Africa, Nigeria has a suicide rate of 17.3 per 100,000, which stands above the global 10.5 and Africa’s 12.0. The rate of suicide is experiencing an exponential increase. As the world, regions, and countries work towards ways to combat the pandemic, scientists brainstorm on preventive modalities. Our team, “The Mending Mind” (Winners of the 2021 Innovation for Action Global Health Challenge) proposed a suicide-preventing innovation that actively works by pathologic stress level detection. The stress-detecting wristband. This innovation is feasible and the technology needed to invent it is available. Moreso, with the rise in Artificial Intelligence (AI) augmented devices, it can be modified over time to include other healthcare monitoring sequences.

## Conference proceedings

The complexity associated with suicide and suicidality is one that global stakeholders in mental health advocacy and therapy have been studying for a while. In recent years, the world seems to now give it more attention. Some traditions viewed suicide as an honorable way to die, having control over your death examples include Seppuku and Harakiri of pre-modern Japan [1]. However, it is now globally accepted as a mental health problem.

No real accurate study effectively estimates the prevalence of suicide, globally. However, the incidence is on the rise. In America, it is the 10^th^ leading cause of death, with over 48,000 deaths annually. Between 2000 and 2018, it increased by 37%. It experienced a decline of 5% afterward up till 2020, and then another spike of 2.7% between 2021 and 2022 [2]. In India, official records estimated 60,000 deaths by suicide in 2021 and over 170,000 deaths by suicide in 2022. That is more than a 100% increase [3,4]. Similarly, the rise in suicide and suicidality has been observed in Iraq, Nigeria, other parts of Africa, Australia, and Asia [5-9]. If we look at the European Union, there were 47,252 deaths due to intentional self-harm reported by Eurostat in 2020, corresponding to approximately 1 out of 100 deaths that year. The countries with more prevalent intentional suicide are Lithuania, Hungary, Slovenia, and Estonia [10].

Socio-demographically speaking, the rate of suicide is more than 2 times higher in men than women [9]. Strikingly, in the 2020 Eurostat report 77.1% of all deaths by self-harm corresponded to men [10]. Yet, it is even higher among men who sleep with men [11]. However, it was discovered that amongst women, it is most commonly related to pregnancy [12]. Amongst the youths, suicide is the 4^th^ leading cause of death in people aged 15-19 years. A study conducted among 12-15-year-old teenagers discovered that most suicide attempts were socially related [13]. This was in keeping with a study in Nigeria that also identified a peak in suicidal ideation in young adults [6]. Although a global study hinted at the possibility of the LGBTQIA+ movement and phenomenon being linked with an increase in suicidality amongst the youth, it did not make conclusive deductions on this [14]. However, this notion was further supported by a quantitative study that deduced that men who sleep with men have a higher rate of suicidality when compared to general heterosexual men [11]. Furthermore, environmental factors such as food insecurity were found to be aggravating factors [15]. Military officers too, but predominantly, the veterans were identified as high risk for Suicidal Ideation (SI) and Suicidal Attempts (SA) [16]. Then in regions with HIV endemicity, it was found as a predisposing factor. Especially amongst viral reactive single parents and women [17].

Generally speaking, one thing that all these predisposing factors have in common is stress. Defining stress has been a headache for psychologists with a great deal of controversy over what the term means. Broadly, stress can be described as the pattern of response an organism makes to stimuli that disturb its equilibrium and tax or exceed its ability to cope. That is, people feel stressed when too much is expected of them, or when events seem scary or worrisome [18-20]. Stressors can be mental, physical, psychological, sociological, financial, or medical stress, among others.

### Link between stress and depression and suicide

These stressors lead to depression, and depression is the major underlying cause of suicide [17]. Depression, characterized by persistent feelings of sadness and loss of interest, is a mood disorder with a multifaceted etiology that remains insufficiently understood [18]. The American Psychiatric Association's Diagnostic and Statistical Manual of Mental Disorders, Fifth Edition (DSM-5), categorizes depressive disorders into several types, including disruptive mood dysregulation disorder, major depressive disorder, persistent depressive disorder (dysthymia), premenstrual dysphoric disorder, and depressive disorder due to another medical condition. Despite their differences, these disorders share common features such as persistent feelings of sadness, emptiness, or irritability, along with somatic and cognitive changes that significantly impair daily functioning [18].

The relationship between stress and depression is multifaceted, characterized by bidirectional causality that can exacerbate both conditions. Understanding the psychological mechanisms underlying this connection is crucial for prevention, particularly for individuals seeking to avert depression relapse following prior episodes. Stress directly impacts mood, manifesting in symptoms such as irritability, sleep disturbances, and cognitive impairments. However, its indirect effects often precipitate depression by disrupting healthy coping strategies. Stress-induced mood changes can lead individuals to forego activities that typically regulate mood, such as exercise or socializing, exacerbating their emotional state. Moreover, initial low mood symptoms can generate further stress, perpetuating a cycle of negative emotions and stressors. Stress also strains relationships, leading to increased conflicts and emotional withdrawal, which further contributes to depressive symptoms. Unhealthy coping mechanisms, such as excessive alcohol use, can compound mood disturbances and exacerbate interpersonal problems, creating a detrimental feedback loop. Additionally, stress disrupts routines and structures, undermining self-regulation and exacerbating mood dysregulation. Recognizing these interconnected pathways can inform targeted interventions aimed at mitigating the deleterious effects of chronic stress on mental health and promoting resilience against depression that can lead to suicide.

The link between depression and suicidal behavior is profound, as evidenced by studies such as the one conducted by Maria Oquendo and colleagues at Columbia University. Their investigation explored the interplay of life events, major depressive episodes, and suicidal behavior over two years among individuals diagnosed with depression. Notably, about 27% of participants also grappled with borderline personality disorder, characterized by emotional dysregulation and impulsivity. Suicidal behavior, defined as self-destructive acts with the intent to end one's life, was prevalent, with approximately 10% of participants reporting such behaviors during the study. Major depressive episodes emerged as the most significant predictor of suicidal behavior, irrespective of the presence of borderline personality disorder [21]. This underscores the critical role of depression in driving suicidal tendencies, highlighting the urgent need for targeted interventions and support for individuals struggling with these mental health challenges.

Every year, more people die as a result of suicide than HIV, malaria, breast cancer, or even war and homicide. Stress and consequently depression are at the leading edge and new efforts to prevent related suicides are needed [22]. While certain countries have prioritized suicide prevention, a considerable number of nations still lack commitment in this area. According to a report by the World Health Organization (WHO) in 2018, there were only 38 countries out of the 194 WHO countries that have established national suicide prevention strategies. That is, 20% of the countries [23]. To achieve the Sustainable Development Goal (SDG) target of reducing the global suicide rate by one-third by 2030, urgent and substantial efforts are required. Suicide prevention can be approached from several levels and modalities. One of the most common methods is therapy. However, the authors of this paper came up with a classification of suicide prevention that will help simplify entry levels (Table 1). In this paper, we also introduce our innovative wristband idea designed to alleviate stress and thereby help prevent suicide.

**Table 1:** the Otobo-Montes' classification of levels of suicide prevention

S/N	Level of prevention	Basic characteristics/group	Intervention/professionals
1	Primary	Non-specific; general population; peers, groups, religious bodies, etc awareness creation	No specific; health promoters; mental health advocates; peers
2	Secondary	Depression; anhedonia; withdrawal; apathy; chronic disease; prolonged grief	Professional psychologist or psychiatrist; family and friends; mentor/respected elder; spouse
3	Tertiary	Suicidal ideation; suicidal attempts	Psychologists and/or psychiatrists; family and friends' support and reassurances
4	Rehabilitative	Suicidal attempts: a) gone ahead with the suicidal action, but was unsuccessful; b) multiple attempts	Psychiatrist/psychotherapist; hospital Admission; restrain; rehabilitation centers.

### The stress-detecting wristband

**Figure 1:**
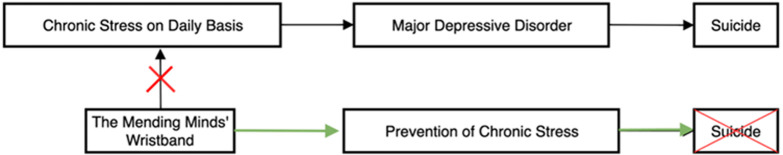
the interplay among chronic stress, major depressive disorder (MDD), suicide, and our proposed solution

Our innovative idea is a wristband to detect stress through innovative technology that will be sensitive and specific enough to measure physical and chemical stressors in the human body such as cortisol and epinephrine (Figure 1). It will be aligned with a complementary app on their mobile phones which will provide insight into symptoms of stress and educational sources on mental health-friendly care for the person. The app will be equipped with a friendly bot that reminds you to destress, as well as check on you from time to time. It will also advise you on your favorite happy places near you with directions, calming song suggestions, and reminders to rest. As an extra, it will also help hypertensive patients monitor and be aware of their blood pressure when it is rising significantly. Figure 1 is a flowchart of the interplay among chronic stress, major depressive disorder (MDD), and suicide and our proposed solution. Chronic daily stress contributes to the onset of major depressive disorder (MDD), heightening the risk of suicide. Our proposed intervention involves a wristband aimed at preventing chronic stress, thereby interrupting the progression toward MDD and potentially reducing stress-related suicide.

### How it works: the science behind it

The wristband detects stress hormone changes in the circulation, heart rates, and body temperature. When the body is under stress, it lights up and sends a signal to the mobile app for more preventive education. This way to a significant extent, the person always has a conscious insight on the state of their mental health. The human body secretes adrenaline when faced with a significant life event that either requires you to fight or fly. This can be seen as an acute stress hormone. In cases of stress and low mood, the body secretes cortisol. This can be seen as a chronic stress hormone. Although there are other significant hormones in depression, like serotonin and dopamine, these cannot be detected cortically, nor do they emit physical parameters that are measurable or detectable cortically.

**Figure 2:**
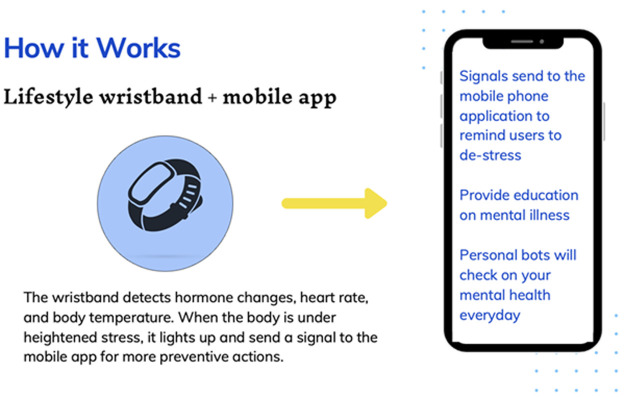
the schematic overview of operation

**Figure 3:**
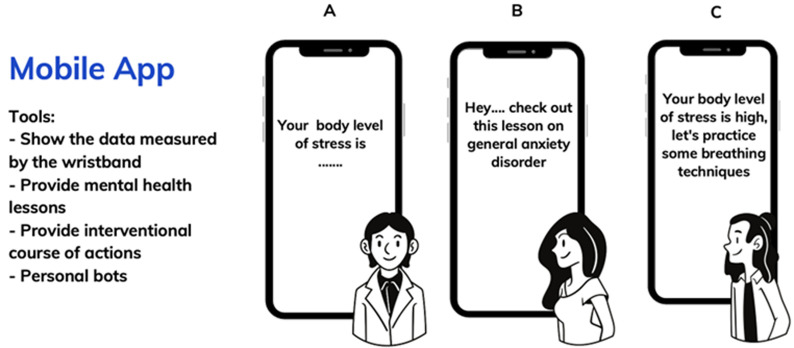
response tools of the mobile application; A) illustration demonstrating the mobile bot app alerting the user of their current stress level; B) seizing an opportunity for educational intervention; C) notifying the user during high-stress periods, prompting them to engage in stress-reduction activities

**Figure 4:**
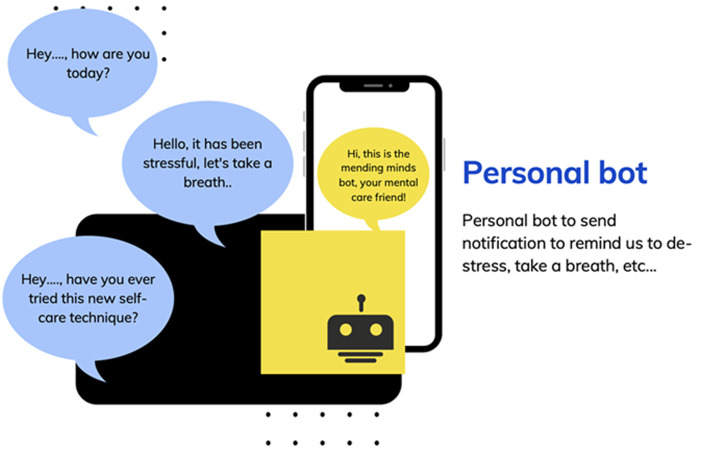
the personal bot; daily check-ins from the mending minds bot app, linked to the wristband

The wristband detects the levels of adrenaline and cortisol, the body temperature, and the pulse (heart) rate. The chemical parameters will be detected via their sweat [23-26]. It will run this against the reference interval for these parameters in its database. If the readings are off, the band lights up and the mobile app alerts you (Figure 1, Figure 2). Figure 3 illustrates a mobile application response tool where the mobile bot app alerts the user of their current stress level (Figure 3A), seizing an opportunity for educational intervention (Figure 3B), and notifying the user during high-stress periods, prompting them to engage in stress-reduction activities (Figure 3C). Figure 4 demonstrates the personal bot, daily check-ins from the mending minds bot app, linked to the wristband, offering quick advice on stress relief, suggesting happy places, providing brief meditation techniques, and offering access to supportive services condensed for easy reading and convenience. This way, we can create a sense of awareness in the general population about their stress levels. Clinically, this can help mental health-associated caregivers and professionals monitor the stress indices of their wards or patients when reviewing the memory of data of the hormonal spikes during clinics. This can be an effective tool to have when caring for prevention level 2 patients (Table 1). Similar to a wearable 24-hour stress ECG (electrocardiography) monitor.

### The long-term effect

In the long term, we will not only be able to prevent poor and fatal mental health conditions such as depression and suicide, but we will also be able to prevent unnecessary weight gain and obesity [27]. Obesity is a medical condition that has a high positive correlation with cardiovascular diseases and diabetes, amongst other chronic conditions [28]. We will have also been able to create not just a mental health insightful individual, but also an advocate. Who will recognize and caution and care for friends and families on stress and depressing life events and how to go about taking care of their mental health.

Lastly, we believe that our idea is unique and should be explored because it is user-friendly, simple, and easy to handle, making it accessible to all. What sets it apart is that it is not just a gadget; it is a mood lifter, cheering up its users whenever they need it as it targets mental care in the everyday lifestyle. Utilizing imputed data and AI-generated memory, it suggests happy places or people for the user to go to or talk to when their stress hormones are spiking. It also includes helplines for the user to reach out to, but automatically reaches out to these helplines when it detects emergency vital signs, such as tachycardia, severe bradycardia, or no cardiovascular activities. Finally, with the aid of an internet source, the app can be launched in a variety of rural and urban areas. Importantly, it has the potential to be used everywhere, even in places where resources are limited such as in the less developed countries, which are frequently the hardest-hit countries by stress-related suicidal rates and mental health issues.

## Conclusion

This is a call to action for collective efforts to destigmatize mental health, provide preventive mental healthcare, raise awareness about physical symptoms of stress, and promote a healthier lifestyle. While our wristband innovation was originally conceptualized for mental healthcare in Bangladesh, a Southeast Asian country where suicide has become a persistent issue. It has the potential to be adapted and implemented globally. By leading the charge in normalizing mental health care, we aim to shine a spotlight on this critical global health challenge that urgently demands attention.
